# Toward improved models of human cancer

**DOI:** 10.1063/5.0030534

**Published:** 2021-01-04

**Authors:** Bryan E. Welm, Christos Vaklavas, Alana L. Welm

**Affiliations:** 1 Department of Surgery, Huntsman Cancer Institute, University of Utah, Salt Lake City, Utah 84112, USA; 2 Department of Internal Medicine, Huntsman Cancer Institute, University of Utah, Salt Lake City, Utah 84112, USA; 3 Department of Oncological Sciences, Huntsman Cancer Institute, University of Utah, Salt Lake City, Utah 84112, USA

## Abstract

Human cancer is a complex and heterogeneous collection of diseases that kills more than 18 million people every year worldwide. Despite advances in detection, diagnosis, and treatments for cancers, new strategies are needed to combat deadly cancers. Models of human cancer continue to evolve for preclinical research and have culminated in patient-derived systems that better represent the diversity and complexity of cancer. Still, no model is perfect. This Perspective attempts to address ways that we can improve the clinical translatability of models used for cancer research, from the point of view of researchers who mainly conduct cancer studies *in vivo*.

Cancer as a global health threat is set to explode, largely due to aging and growth of the population. According to the latest edition of The Cancer Atlas, produced by the International Agency for Research on Cancer, it is anticipated that there will be 29 million cancer diagnoses in 2040, compared to the already astonishing 18 million cases in 2018 (https://canceratlas.cancer.org). This is alarming because, despite efforts to improve cancer prevention and treatment, many cancers are still deadly. As of 2018, cancer is the cause of death for 9% of all women and 13% of all men. Remarkably, cancer ranks as the first or second cause of premature death in 134 countries across the world.

New approaches to diagnose and treat deadly cancers will be essential to curbing the growing impact of this disease. We emphasize deadly cancers here because more sophisticated and widely implemented screening will surely lead to increased diagnosis of cancer. A major challenge is, and will continue to be, distinguishing indolent cancers from deadly ones so as to avoid incurring toxicities and other costs by aggressively treating tumors that are unlikely to progress. It goes without saying, however, that new therapies are also desperately needed to treat deadly cancers in an effective manner. Here, as researchers primarily working with *in vivo* models of cancer, we focus on new developments and ongoing needs in human cancer research, focusing on how tissue donations and development of patient-derived models are showing promise for improving research in cancer diagnosis, prognosis, and treatment.

Cancer, as a complex biological process, requires extensive use of models in order to effectively delineate its biology and to ethically test new therapies. Thomas *et al.* recently reviewed the history of cancer models,^1^ nicely emphasizing the iterative process of model development, testing, and improvement. Specifically, one must first identify the question or problem to be solved. Next, one needs to build the model(s) by identifying an experimental system that can be manipulated to test the hypothesis. The model is then tested, and outcomes are evaluated. Outcomes must be compared to those already known and to other available data, such as clinical evidence. Evaluation of the clinical relevance is critical to model improvement, as models without clinical relevance have little practical value.^1^ Thus, *evolution* of models is necessary to apply the newest technologies and to integrate new knowledge. As cancer models have evolved from early animal models to test carcinogenic effects of various substances, then to human cancer cell lines grown in culture, and on to genetically engineered mouse models and human tumor xenografts in various hosts, we have gained valuable knowledge about cancer biology. Information gained from these models, and the use of models for pre-clinical therapeutic studies, has supported the development of effective new treatments for cancer. Yet, we are still struggling to succeed with the discovery of therapies that really curb cancer deaths. We believe these failures are largely due to shortcomings in our preclinical models.

In contriving model systems, prior knowledge must be used to develop the model. For example, mice can be genetically engineered to test the function of a candidate oncogene or tumor suppressor, and the functions of specific genes or signaling pathways can be elucidated in human cancer cell lines. However, contrived models carry the risk of “streetlight bias,” because they implement and investigate the variables for which we have knowledge, but limit inquiry into elements that remain in the dark. All models are manufactured to some extent, and the more they are engineered, the greater the potential for streetlight bias. State-of-the-art *in vitro* culture models, for instance, might integrate several important cell types and extracellular matrices, and even simulate blood flow, to recapitulate heterogeneous tumors and build something akin to an organ-on-a-chip.^2^ Such a system is conducive to high-throughput drug screening, but may miss critical physiology not yet discovered or not amenable to the culture setting. The biology of tumors, including drug responses, can be greatly influenced by culture conditions. For example, the simple manipulation of extracellular matrix:tumor interactions was sufficient to completely revert the malignant phenotype of breast cancer cells *in vitro*,^3^ and differences in HER2 signaling and trastuzumab efficacy were shown when cells were grown in 2D vs 3D cultures.^4^ *In vitro* models may also bias toward the use of models that are more amenable to culture conditions or may oversimplify complicated processes like invasion and metastasis.^5^

On the other hand, *in vivo* animal models of cancer allow many unknown processes to occur alongside the known variables. For example, blood vessels develop in response to the hypoxic nature of tumors but the exact patterning and regulation of their function are still not understood;^6^ immune cells and other stromal components infiltrate tumors according to signals yet to be defined;^7^ metastasis proceeds throughout complex organ systems through many unknown mechanisms;^8^ and drugs can be tested for efficacy without knowing how many stromal cell types or other signals contribute to the therapeutic response.^9^ All of these processes occur in, for example, mouse models of cancer—even if we don't understand how to recapitulate them *ex vivo*. Still, by their very nature, models are simply attempts to emulate human cancers in the absence of our ability to fully replicate the disease for experimentation. No model is perfect. So which models carry the greatest relevance for human tumors?

Perhaps one of the most enlightening recent discoveries in human cancer, made possible with the advancement of next-generation DNA sequencing technology, is the striking heterogeneity of tumors. Identification of genetic alterations that are shared across large numbers of tumors raised hope that common genetic “drivers” could be identified and targeted for therapeutic benefit. Unfortunately, precision oncology based on genomic mutations has not proven to be the silver bullet we had hoped for.^10,11^ While some cancers exhibit a bias for specific mutations, others are vastly different between individuals and are even divergent between multiple tumors in the same individual (e.g., primary tumors and metastatic lesions). Furthermore, as cancers evolve during patient treatment, resistance mechanisms are predictable in some instances, but in other cases, resistance is achieved through diverse or unknown pathways. Thus, as standard-of-care therapies evolve, so do cancers, and representative models must be developed for each clinical era. Just because a cancer cell line or co-cultured fibroblasts came from a human donor at some point does not mean the resulting model is really representative of the disease one is trying to study.

The diversity of human tumors, along with a long history of cancer drug failures,^12^ has prompted renewed interest in generating patient-derived models for cancer research (Fig. 1). Modern patient-derived models are an attempt to represent bona fide human tumors that evolve under current therapies and can be grown in various conditions, most commonly as patient-derived xenografts (PDX) in mice or in three-dimensional (3D) organoid cultures. Studies have shown that patient-derived models, in general, recapitulate much of the heterogeneity of the original tumors and accurately model treatment responses in the patients from which they are derived—at least better than previous attempts with cancer cell lines.^13^ Remarkably, for some cancer types, the ability to engraft patient tumors as PDX in mice also gives independent prognostic information, because only the most aggressive tumors successfully engraft.^14,15^ As a result, PDX models are being used not only for prognostic purposes and preclinical drug testing but also to understand heterogeneity and evolution of human tumors, and the complicated process of metastasis.^14,15^ Such models are not without caveats, however, including lack of a functional immune system unless “immune-humanized” mice are used (the utility of which is still debatable^16^) and lack of other species-specific signaling components.^13^ The time required to carry out experiments *in vivo* and the prohibitive cost are also barriers to discovery research using PDX models.

**FIG. 1.**
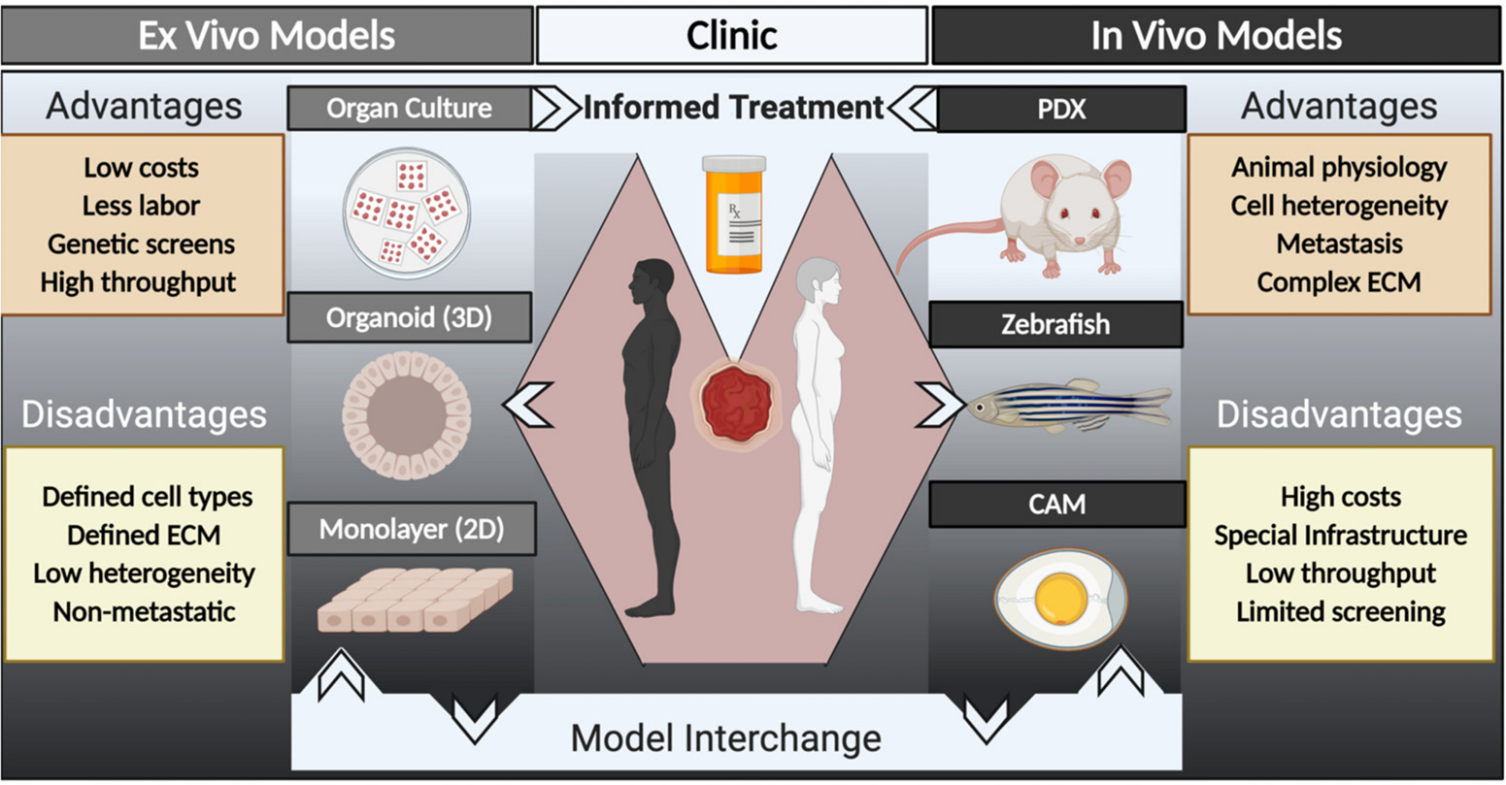
Schematic illustrating multiple platforms available for patient-derived models of cancer. *Ex vivo* and *in vitro* models (left side) include organ or tissue slice cultures, three-dimensional (3D) organoids, and two-dimensional (2D) monolayer cultures. *In vivo* models (right side) include patient-derived xenografts (PDX) in mouse, zebrafish, and chicken egg chorioallantoic membrane (CAM) hosts. Information from these model systems can be interchanged to increase our understanding of human cancer biology, to perform drug screening or testing, and, in some cases, for functional precision oncology to inform patient care.

Generation of “simpler” patient-derived models such as tumor spheroids and organoids, which also retain high fidelity to patient tumors,^17,18^ overcomes some of the barriers of *in vivo* PDX models. These 3D cell culture systems can be grown short-term or long-term, allow larger scale investigation, such as drug screening or CRISPR/Cas9-mediated genetic manipulation,^19^ and, when developed side-by-side with PDX models from the same tumor, allow interchangeable *in vivo* and *in vitro* investigation. Co-culture systems (with the same caveats discussed above) can be developed to study heterotypic cell interactions such as immune responses,^20^ and effects of controlled microenvironments on tumor behavior can be tested.^21^ Patient-derived models of cancer have been also extended to include PDX in zebrafish embryos^22–25^ and on chicken egg chorioallantoic membranes (CAM)^26^ for efficient drug testing while retaining an *in vivo* setting. Similar to the discussion above for mouse PDX models, these alternative *in vivo* patient-derived models have advantages of a more physiologically relevant, whole-body microenvironment than *in vitro* models, but any xenograft has the caveat of tumors being grown in a foreign, typically immune-deficient (or suppressed), host. Thus, interchanging a combination of various types of patient-derived models (Fig. 1) may be the most fruitful way to model human cancer to date.

There is still a paucity of appropriate models, however, for some of the most urgent questions in cancer biology. If we really want to make a difference in cancer mortality, we need to build models that are clinically relevant, and care should be taken to mimic the intended application as closely as possible. For example, for studies to prevent metastasis, spontaneously metastatic models should be utilized, at least in combination with experimental metastasis models. For investigation of metastatic cancer and development of new therapies to be used in the metastatic setting, one should utilize models made from metastatic lesions, not those made from primary tumors, despite the fact that the latter samples are more readily available. Models from metastatic lesions should be made from tissues that have been exposed to today's treatments, not those from decades ago when many of the most commonly used cancer cell lines were developed. To examine mechanisms of drug resistance, one should utilize models made from treatment-resistant tumors, not from therapy naïve ones. Rapid autopsy specimens, although challenging to coordinate and collect, are crucial for developing models from end stage cancers for studies of inter- and intra-tumor heterogeneities and drug resistance.

What does the future hold for human cancer modeling? We believe we are on the cusp of a true paradigm shift: a convergence of sophisticated patient-derived models, powerful co-clinical trials (discussed below), and formidable advances in machine learning. Patient-derived models are not only being used for standard research and development; they are now being used as tools for *functional* precision oncology. In the latter case, tumor samples from individual patients are tested for susceptibility to various drugs in the context of a PDX^27,28^ or organoid culture,^29,30^ in order to inform patient therapy during the course of their disease. Such an approach allows functional assessment of drug response and is not limited to mutation analysis, which exemplifies most current precision oncology strategies.^31^ While the functional precision oncology approach is not currently feasible as standard care for the masses, it is instructive to validate and evolve our models based on concordance between patient and model drug responses and for comparison of tumor heterogeneity and evolution.

An even more powerful approach to human cancer modeling would be to facilitate co-clinical trials: simply coupling patient-derived model development to the plethora of clinical trials being run every day, as a new standard procedure. Tumor biopsies are often taken prior to treatment on clinical trials and often again upon progression of disease, but models are almost never made from these samples! At a minimum, simple preservation of viable samples should be as routine as preparing fixed or flash frozen tissue. Given the importance of therapy-induced evolution following treatment, imagine if one had the ability to use viable models collected from pivotal clinical trials to investigate response or resistance to the investigational drug, and tumor evolution following progression, in a co-clinical setting. At the completion of the trial, models would be available to immediately begin mechanistic experiments to determine why responders were responders or why certain tumors were resistant. One could use these very models to determine how to overcome drug resistance with the next therapy; thus, models representing the next clinical era would be available *ahead of the curve*.

As a future Perspective, if the above-mentioned co-clinical data and models were collated and shared, machine learning may facilitate “big data” analysis to discover complex patterns of drug response or resistance across individuals, which could then be tested in the patient-derived models. Indeed, such computational resources are being developed^32^ and hold great promise for drug response prediction. Public access to deidentified data (specifically, tumor phenotype and genotype data along with drug response information) would allow this field to move forward more quickly, and more effectively address the urgent medical needs in cancer treatment. At some point in the future, we envision that patients' tumors may be bioinformatically profiled to identify a complex set of features predictive of response to various therapies, informed by functional drug response data collated from previous studies. This would allow early selection of more effective drugs while preventing administration of toxic drugs with no benefit. This type of data could even be integrated with germline DNA sequence variants that predict aberrant drug metabolism and toxicity for an even more personalized approach^33^ to reduce mortality from cancer while simultaneously reducing toxicity as much as possible. Thus, the future is poised for new approaches that will meaningfully reduce cancer mortality, but it will require that we all take extra steps to generate and utilize the best models possible for discovery and validation of cancer mechanisms and therapies.

## Data Availability

Data sharing is not applicable to this article as no new data were created or analyzed in this study.
